# Calculation of blood loss in cardiac surgery: How should we monitor?

**DOI:** 10.1177/02676591251370110

**Published:** 2025-08-20

**Authors:** Yerlan Orazymbetov, Serik Aitaliyev, Povilas Jakuška, Audronė Veikutienė, Tadas Lenkutis, Rassul Zhumagaliyev, Aušra Saudargienė, Rimantas Benetis

**Affiliations:** 1 Department of Cardiac, Thoracic and Vascular Surgery, 369695 Hospital of Lithuanian University of Health Sciences Kauno Klinikos, Medical Academy, Lithuanian University of Health Sciences, Kaunas, Lithuania; 2 Department of Cardiac Surgery, National Scientific Medical Centre, Astana, Kazakhstan; 3 Faculty of Medicine and Health Care, Al-Farabi Kazakh National University, Almaty, Kazakhstan; 4 Institute of Cardiology, Medical Academy, 369695 Lithuanian University of Health Sciences, Kaunas, Lithuania; 5 Department of Anaesthesiology, Medical Academy, 369695 Lithuanian University of Health Sciences, Kaunas, Lithuania; 6 Neuroscience Institute, 369695 Lithuanian University of Health Sciences, Kaunas, Lithuania

**Keywords:** blood loss, Hb/kg index, chest tubes, body mass, cardiac surgery

## Abstract

**Background:**

The bleeding in cardiac surgery remains a significant clinical problem. There is no “gold standard” method to quantify blood loss. Traditional measurement of drainage volume often underestimates or overestimates, as it does not consider the type of fluid. We hypothesized that blood loss could be more accurately calculated using the Hb/kg index in terms of haemoglobin (Hb) mass loss per kilogram of the patient’s body mass.

**This study aimed to Objective:**

To develop a novel approach for calculating actual blood loss using the Hb/kg index.

**Methods:**

This single-center prospective study included 195 patients who underwent cardiac surgery between October 2023 and November 2024. The Hb/kg index was calculated based on intraoperative Hb loss, Hb loss via chest tubes, packed red blood cell transfusions and patient weight. Eighty-six additional clinical predictors were analyzed using conventional statistics and machine learning algorithms. Predictors with statistically significant Spearman correlations were included for further analysis.

**Results:**

Lasso regression achieved the best overall performance in predicting Hb/kg index. It yielded the lowest mean squared error (0.08 ± 0.04), mean absolute percentage error (0.18 ± 0.10), with the highest correlation (0.92 ± 0.06) and R² score (0.82 ± 0.13). BMI showed a significant negative relationship (−0.018, *p* < 0.001). Postoperative Hb and haematocrit values had negative correlation (−0.69, *p* < 0.001 and −0.07, *p* < 0.015), while initial Hb was positively correlated (0.85, *p* < 0.001).

**Conclusions:**

This method provides a more reliable and clinically relevant tool to calculate actual blood loss and allows for a more precise assessment and treatment.

## Introduction

The bleeding is still significant clinical challenge in cardiac surgery.^1,2^ Excessive bleeding increases the rate of reoperations, the need for blood transfusion, the length of hospital stay and costs.^3^ Re-exploration due to bleeding is an independent predictor of adverse outcomes, including stroke, sternal wound infection and hemodialysis. It is also associated with higher morbidity and mortality.^4^ While the average blood loss after heart surgery is about 300 mL, up to 10% of patients experience excessive bleeding,^5^ and 3%–7% require a re-sternotomy to stop the bleeding.^6–10^ A study of 798 surgical departments in the United States found that the percentage of patients requiring a blood transfusion ranged from 8% to 93%.^11^ Despite its clinical relevance, there is no “gold standard” method for quantifying actual blood loss.^12^ Traditional methods are based on drainage volume measurement, which can be inaccurate as they can be under- or overestimated.^13^ Some authors defined active bleeding as a blood loss exceeding 1.5 mL/kg/hour for six consecutive hours,^14^ while others suggested a blood loss of more than 200 mL within 1 h.^15^ However, none of these definitions mention the type of fluid drained via the chest tube drainage (CTD). This is particularly important because the fluid drained via CTD may consist of hemorrhagic, serous, or a combination of both.^16^ Another important question is how the same amount of blood loss affects hemodynamics and postoperative complications in patients with different body mass. People with a higher body mass index (BMI) have a larger estimated blood volume (EBV) and can tolerate a certain amount of blood loss better than people with a lower BMI and vice versa.^17,18^

Considering Hb mass loss via chest tubes, as well as the effects of red blood cell transfusion (PRBC) on Hb levels and body weight, we hypothesized that calculating blood loss based on the Hb/kg index (in terms of Hb mass loss per kilogram) is a more accurate method of calculation of blood loss.

## Materials and methods

### Study design and population

This prospective observational study was conducted at the Department of Cardiac, Thoracic and Vascular Surgery, Hospital of Lithuanian University of Health Sciences, Kaunas, Lithuania, between October 2023 and November 2024. A total of 195 adult patients undergoing elective cardiac surgery with CPB were included. Exclusion criteria were urgent or redo procedures, re-sternotomy for bleeding or tamponade, aortic dissection, heart transplantation, mechanical assist device implantation, hematologic disorders, and liver or kidney failure. The data were collected at the following time points: T_0_ (preoperative), T_1_ (immediately postoperative), T_6_ and T_18_ (6 and 18 h postoperative). CTD volumes were recorded hourly from 1 to 18 h after surgery (CTD1h–CTD 18 h).

### Ethical approval

The study was approved by the Kaunas Regional Bioethics Committee (No. BE-2-53, August 2023). All patients provided written informed consent prior to participation.

### Data collection

The following data were collected: age, gender, BMI, estimated blood volume (EBV), type of surgery, number of grafts, coagulation parameters (PT, aPTT, INR, fibrinogen, D-dimer). Hb-related parameters were determined at different time points using samples from the chest tube and the central venous catheter. No patient received antifibrinolytic therapy within 5–7 days prior to surgery. All procedures were performed under general anaesthesia and CPB according to standard protocols. The follow-up period continued until discharge from the hospital.

### Statistical analysis

The data were analyzed using using the sci-kit-learn Python package (version 3.2), with Keras Python Framework^19^ and IMB SPSS Statistics version 30.0 (SPSS Inc., Chicago, IL, USA). The Hb/kg index was compared across groups by gender, BMI (Group I: <24.9 kg/m^2^, Group II: 25–29.9 kg/m^2^ and Group III: ≥30 kg/m^2^), type of surgery (Group I: coronary artery bypass grafting, Group II: valve surgery and Group III: combined surgery) and CTD volume (Group I: <500 mL, Group II ≥500 mL) using independent t-tests and one-way ANOVA. Correlation with age was evaluated using Pearsons coefficient. To identify independent predictors of blood loss (Hb/kg index), 86 variables were analyzed using both conventional statistical methods and machine learning algorithms, including Linear Regression (LR), Lasso Regression, Lasso Regression combined with Ordinary Least Squares (Lasso-OLS), Support Vector Machine (SVM), k-Nearest Neighbors (kNN), Decision Tree (DT), Extreme Gradient Boosting (XGBoost) and Deep Neural Networks (DNN) (See full statistical analysis in Supplemental material).

### Hb/kg index calculation

The index was calculated in five steps using formulas and clinical data:

#### Estimated blood volume

Several formulas exist in the literature to estimate a patient’s blood volume,^20–22^

EBV was calculated using the Nadler formula, which includes height, weight and sex-specific coefficients.^21^ This method provides high accuracy for all body types compared to other methods, such as the International Council for Standardisation in Haematology (ICSH formula), which is based on body surface area.^22^

$$EBV\left(L\right)=k_{1}\times Height{\left(m\right)}^{3}+k_{2}\times Weight\left(kg\right)+k_{3};$$

where:

• For men: k_1_ = 0.3669, k_2_ = 0.03219, k_3_ = 0.6041;
• For women: k_1_ = 0.3561, k_2_ = 0.03308, k_3_ = 0.1833.

#### Initial Hb mass

The initial Hb mass was calculated by multiplying the haemoglobin concentration (g/L) by the EBV:

$$Initial\;Hb\;mass=EBV\left(L\right)\times {Hb}_{concentration}\;\left(g/L\right)$$

#### Intraoperative Hb mass loss

Intraoperative Hb loss was calculated using the difference in Hb mass before and after surgery, adjusted for EBV^23^:

Hb mass loss (intra−op)=(Initial Hb mass – Hb mass (post−op))*EBV

#### Hb mass loss via CTD

CTD was recorded within 18 hours postoperatively. Fluid from chest tube was continuously collected and measured in the reservoir, which was connected to the vacuum system (GENTEC Model 882VR Continuous Suction Regulators, CA, USA). Aliquots of the drained fluid were sent to our hospital laboratory where the haemoglobin concentration (g/L) was determined using automated haematology analyser. The total haemoglobin mass in the chest tube was then determined by multiplying the haemoglobin concentration to the CTD volume:

$$Hb\;loss\;\left(CTD\right)={Hb}_{concentration}\left(CTD\right)\times Total\;volume\;\left(CTD\right)$$

#### PRBC transfusion

In general, one unit of PRBC increases the Hb concentration by about 10 g/L.^24^ According to Lisander et al. one unit of PRBC contains about 52 ± 5.4 g Hb.^25^ In our case, however, we found that one unit of PRBC contains at least 43 g of hemoglobin.

Hb transfusion (g)=Number of PRBC units×43 g

After calculating the total Hb mass loss, this value is subtracted from the patient’s initial Hb and divided by the patients body weight. Thus, the empirical formula can be optimized and presented as follows:

$$Hb/kg\;index=\frac{Hb\;loss\;\left(intra-op\right)+Hb\;loss\;\left(CTD\right)-Hb\;transfusion\;\left(PRBC\right)\;}{Body\;weight\;\left(kg\right)}$$

## Results

A total of 212 patients undergoing elective cardiac surgery with CPB were included. 17 were excluded: one (0.5%) withdrew, four (1.9%) had incomplete data, and twelve (5.7%) required re-sternotomy for bleeding or tamponade. No in-hospital mortality was observed. The final cohort included 195 patients (134 men and 61 women) with a mean age 66 ± 7 years. Comorbidities included type 2 diabetes in 15.9%, hypertension in 40.5% and hyperlipidemia in 83.1%. According to the NYHA classification, 60% were classified as class I–II and 40% as class III–IV. Surgical procedures included CABG (51.8%), valve surgery (29.2%) and combined surgery (19%). The number of grafts was distributed as follows: none (28.7%), 1 graft (4.6%), 2 grafts (9.2%), 3 grafts (37.4%), and ≥4 grafts (20%). The valve procedures included a range of interventions such as aortic valve replacement (including both mechanical and biological prostheses), aortic valve repair, ascending aorta replacement, tricuspid valve repair, mitral valve repair and mitral valve replacement. PRBC transfusions were given only when Hb <80 g/L. No other blood components (FFP, platelets, cryoprecipitate) were routinely used. PRBC transfusion was administered to 14 patients (7.2%): 11 received 1 unit and 3 received 2 units intraoperatively or within 18 h postoperatively. Transfusion was more frequent in women (13%) than men (4.5%). The differences in Hb/kg index between genders, obesity, surgery type, CTD groups and age are shown in (See Table 1 in Supplemental material). Men had a significantly higher mean index than women (1.98 ± 0.69 vs 1.76 ± 0.76; t (193) = 2.031, *p* = 0.044). The mean age of the patients was 65.8 ± 7.1 years, with no significant correlation between age and Hb/kg index (r, *p* = 0.628). The Hb/kg index differed significantly between the BMI groups: Group I (n = 56) had a mean of 2.08 ± 0.73, Group II (n = 67) 2.06 ± 0.71 and Group III (n = 72) 1.64 ± 0.64 (ANOVA F (2,192) = 8.367, *p* < 0.001, η^2^ = 0.08). The Scheffé test revealed a significant difference between groups I and III (p = 0.003), indicating higher blood loss in patients with a lower BMI. Type of surgery showed no statistically significant difference (F (2,192) = 1.436, *p* = 0.857). Moreover, both CPB and aortic cross-clamp time showed no significant correlation with the Hb/kg index. CTD Group II (n = 50) had a higher index (2.11 ± 0.68) than CTD Group I (n = 145, 1.84 ± 0.72; *t* (193) = −2.88, *p* = 0.021). Patients were stratified into two CTD‐volume groups based on total chest-tube output over the first 18 h after surgery: Group I (<500 mL) and Group II (≥500 mL). The differences in Hb/kg Index and CTD groups are shown in Table 1). The predictive features selected by Lasso regression demonstrated the strongest positive correlation with intraoperative hemoglobin loss (ρ = 0.82, *p* < 0.001), followed by initial hemoglobin concentration (ρ = 0.37, *p* < 0.001). The strongest negative correlations were observed with BMI (ρ = −0.31, *p* < 0.001) and PRBC transfusion (ρ = −0.29, *p* < 0.001) (See Table 2 in Supplemental material). Spearman correlation coefficients (ρ) were calculated for 86 predictors (See Table 3 in Supplemental material). The variables characterizing Hb and RBC levels at different time points also showed significant negative correlations, underlining their dynamic importance. Fibrinogen levels at T_1_ (ρ = −0.31, *p* < 0.001), T6 (ρ = −0.19, *p* = 0.009) and T18 (ρ = −0.28, *p* < 0.001) showed moderate negative correlations. CTD variables had weak, but significant positive correlations, especially for CTD_5h_ (ρ = 0.23) and CTD_6h_ (ρ = 0.24; both *p* = 0.001).

**Figure 1.**
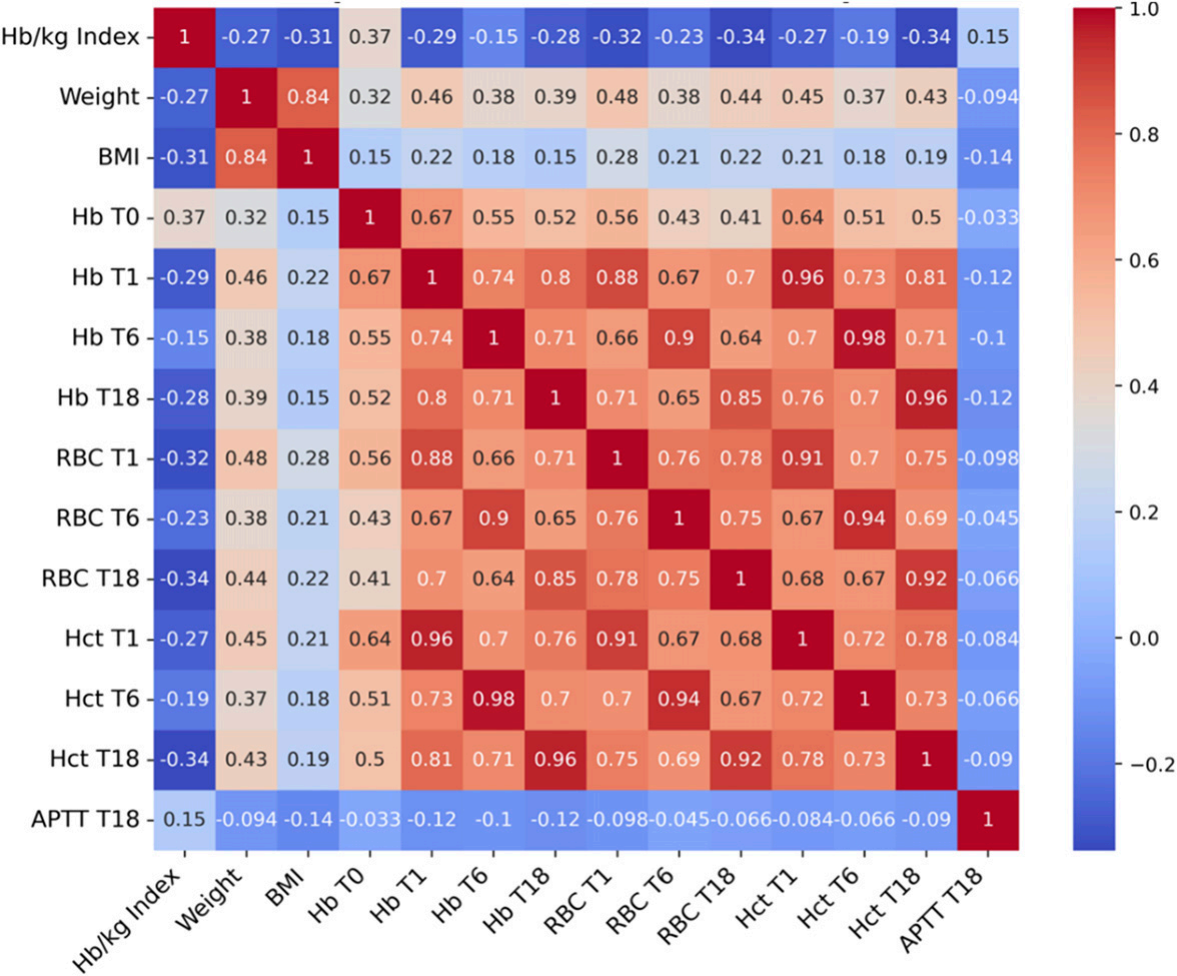
Spearman correlation coefficients illustrating the association between the Hb/kg index and complete blood count (Hb, RBC, Hct) and aPTT.

**Figure 2.**
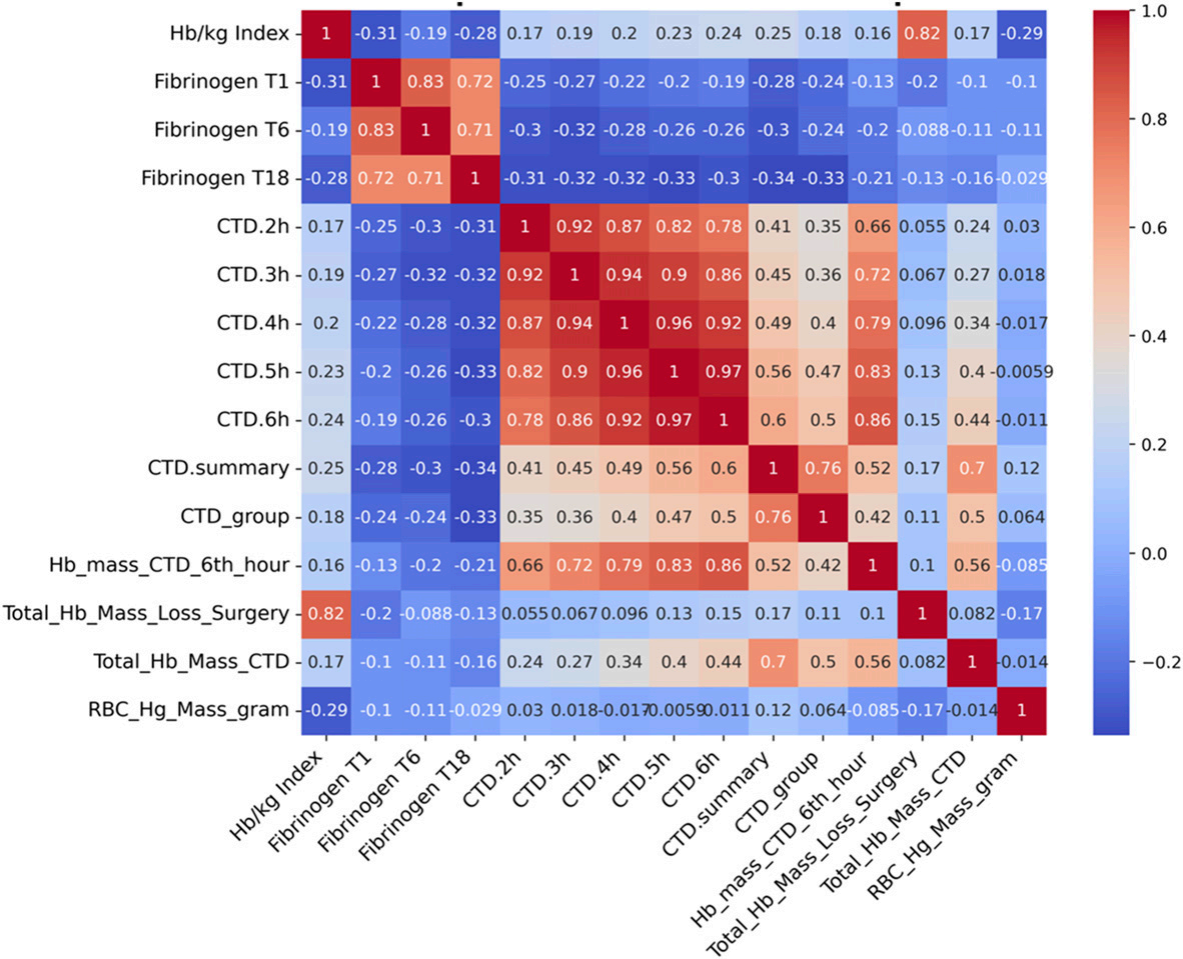
Spearman correlation coefficients illustrating the association between the Hb/kg index and selected variables (fibrinogen levels, chest tube drainage volumes, and hemoglobin mass loss).

The correlation matrix, which illustrates the strength of the relationships between the variables and the Hb/kg index is shown in Figures 1 and 2.

**Figure 3.**
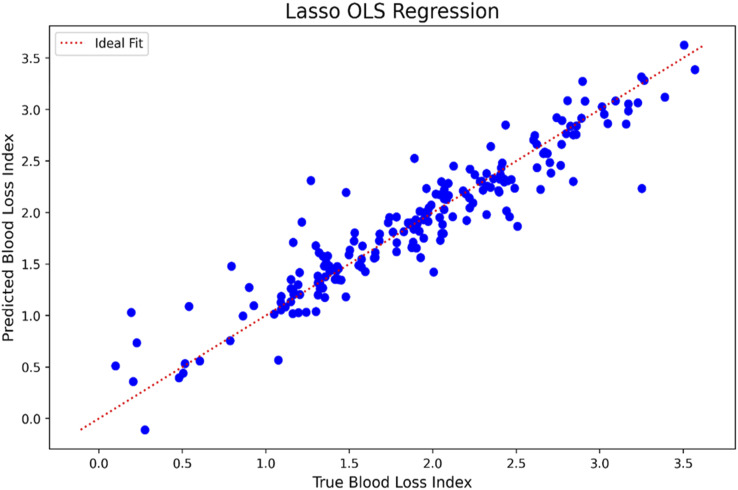
Predicted versus true blood loss index using Lasso OLS regression on the full data set.

Predictors with statistically significant Spearman correlations were used in the machine learning models, with the exception of Lasso and Lasso-OLS, which performed internal feature selection. Among all models, Lasso regression showed the best performance with the lowest mean squared error (0.08 ± 0.04), mean absolute percentage error (0.18 ± 0.10), and highest correlation with the target variable (0.92 ± 0.06) and R^2^ (0.82 ± 0.13) (See Table 4 in Supplemental material). The traditional linear regression also performed well. XGBoost and SVM showed comparable accuracy, while k-nearest neighbors, decision tree and neural network models performed below average (e.g. kNN: correlation 0.58; R^2^ = 0.26). The strong performance of Lasso suggests that the Hb/kg index follows a predominantly linear relationship that is optimally captured by a sparse feature set (Figure 3). Each blue dot represents one observation. The red dotted line indicates the ideal prediction. The close clustering of points around the line suggests good model performance.

## Discussion

All cardiac surgery patients are at risk of bleeding.^26^ Numerous methods for calculating blood loss have been described in the literature,^12,13,26–28^ but none of them is a generally accepted as a “gold standard”.^13,26^ Conventional estimation methods are based on volumetric or visual assessments^27^ are known to be inaccurate and subjective, often leading to under- or overestimation.^29^ For example, Tran et al. showed that visual estimation significantly underestimated blood loss,^30^ while Lin et al. found that the visual method tended to provide lower blood loss volumes than the formula-based method.^31^ Several formulas have been proposed for estimating blood loss,^12,13,28,32^ but they do not calculate Hb mass loss via chest tube or its relationship to the patient’s body weight.

In cardiac surgery, chest tubes are routinely used to prevent complications caused by the accumulation of blood or serous fluid in the chest. The quality and status of postoperative hemostasis and the detection of bleeding are often monitored by the volume and character of chest tube output.^33^ Christensen et al. reported that postoperative bleeding of more than 200 mL/hour within the first 6 h after cardiac surgery is significantly associated with increased mortality.^15^ Similarly, Colson et al. defined active bleeding after cardiac surgery as blood loss of more than 1.5 mL/kg/h over six consecutive hours within the first 24 h.^14^ However, several studies demonstrated the inaccuracy of visual estimation methods. Measuring only the hemoglobin concentration in the drainage does not account for dilution by irrigation fluids and serous exudate, leading to inaccurate estimates.^15^ Gerdessen et al. illustrate this overestimation due to fluid dilution, reporting an estimated mean blood loss of 500 mL compared to an actual mean of 281.5 mL.^34^ In our study, patients were divided into two groups based on CTD volume: Group I (<500 mL) and Group II (≥500 mL). Group II had a significantly higher Hb/kg index compared to group I. These results show a significant positive correlation, underlining the clinical relevance of the Hb/kg index as a potential predictive marker for calculating blood loss. An important consideration is that the same amount of blood loss can have different physiological effects depending on the patient’s body weight. The effects of BMI on bleeding risk and short- and long-term outcomes have not been adequately researched, and few data are available in the literature. Gutierrez et al. suggested that individuals with a higher BMI generally have a larger estimated blood volume, which allows for better physiological tolerance to blood loss than individuals with a lower BMI.^17^ Bhavsar et al. found in a large cohort study of 12,606 patients undergoing coronary artery bypass grafting (CABG) that obese patients (BMI ≥40 kg/m^2^) had significantly less postoperative bleeding and required fewer blood transfusions.^18^ In contrast, underweight patients (BMI <18.5 kg/m^2^) had a higher risk of excessive bleeding and need for blood transfusions. The authors emphasized the influence of BMI on surgical outcomes and advocated individualized perioperative strategies based on BMI categories.^17,18^ Our results support previous findings showing that underweight patients are at increased risk of postoperative bleeding and transfusion requirements, while obese patients may tolerate blood loss more effectively due to greater EBV. In fact, between 27% and 92% of patients undergoing cardiac surgery receive at least one unit of PRBCs,^11,35^ which is associated with increased morbidity and mortality.^36^ Hb mass transfusion (PRBC) is an important confounding factor in the calculation of Hb mass loss. Lisander et al. reported that one unit of stored blood contains 52 ± 5.4 g Hb.^25^ We have determined that one unit of PRBC should provide approximately 43 g of hemoglobin, based on national standards for blood products. Our results show that PRBC transfusion was a significant negative predictor of Hb/kg index, with moderate negative correlations observed.

In this study, we developed an empirical formula for calculating actual blood loss based on the Hb/kg index. The model was developed using both traditional statistical methods and machine learning algorithms. Among the regression models evaluated, Lasso regression demonstrated the best performance in predicting the Hb/kg index. These findings suggest that the Hb/kg index can be effectively modeled using a sparse linear approach that emphasizes the most clinically relevant features.

Given the critical need for a more accurate and physiologically relevant method to stratify bleeding severity in cardiac surgery, the comprehensive validation and detailed analysis necessary for its full clinical significance will demand further extensive prospective data collection and rigorous statistical modeling. To ensure the robustness and clinical applicability of this concept, a complete demonstration alongside a thorough investigation of its clinical correlates and outcomes will be an important goal for future research.

## Conclusion

In this study, using the Hb/kg index to calculate blood loss might be more accurate than traditional volume-based methods. Although this approach has the potential to be a valuable research tool, further studies are needed to confirm its reliability.

## Supplemental Material

Supplemental Material - A prospective randomised comparative study of dynamic, static progressive and serial static proximal interphalangeal joint extension orthoses Supplemental Material for Calculation of blood loss in cardiac surgery: How should we monitor? by Yerlan Orazymbetov, Serik Aitaliyev, Povilas Jakuška, Audronė Veikutienė, Tadas Lenkutis, Rassul Zhumagaliyev, Aušra Saudargienė and Rimantas Benetis in Perfusion
