# Supplementary material for: Calculation of blood loss in cardiac surgery: How should we monitor?
Source: Perfusion. 2025 Aug 20;41(4):470–7. doi: 10.1177/02676591251370110 (PMC13144624; doi:10.1177/02676591251370110)
Supplement: Supplemental Material - A prospective randomised comparative study of dynamic, static progressive and serial static proximal interphalangeal joint extension orthoses [file sj-pdf-1-prf-10.1177_02676591251370110.pdf]

Statistical analysis.

Spearman's rank correlation coefficients were calculated between the predictors and the Hb/kg index, as the variables were not normally distributed. Variables with statistically significant correlations ($p < 0.05$) were included in further analyses, except in the Lasso and Lasso-OLS models. Independent variables with absolute skewness greater than 2 were log-transformed to correct for skewness and meet the assumptions of normality and constant variance in the Linear Regression and Lasso models.

The LR model was used for feature selection and prediction using L1 regularization. It was trained on the input data with an alpha of 0.1 to control for the strength of regularization. Predictors with non-zero coefficients from the lasso model were then used in an Ordinary Least Squares (OLS) regression model to estimate coefficients and p-values. The performance of the OLS model was evaluated using the same accuracy metrics. This combined Lasso-OLS approach provided both predictive accuracy and model interpretability.

The SVM model used a Radial Basis Function (RBF) kernel, with a regularization parameter of $C = 10$ to penalize large deviations and an epsilon (ε) value of 0.1 to define the tolerance range. The gamma parameter was automatically calculated based on data variance.

The k-Nearest Neighbors (kNN) model used 8 neighbors and the Euclidean distance metric.

The XGBoost model was built as an ensemble of 100 decision trees, with each tree trained sequentially to correct the errors of the previous ones. The learning rate was 0.1, the maximum tree depth was 3, and both the subsample and feature fraction were set to 0.8, meaning that 80% of the data and features were randomly selected for each tree.

The Neural network model (DNN) was a fully connected feedforward network with three hidden layers of decreasing size. The first layer had 40 neurons with ReLU activation, followed by a dropout layer with a 30% dropout rate to reduce overfitting. The second layer included 15 neurons with ReLU activation and a 20% dropout rate, and the third layer had 5 neurons with ReLU activation and 20% dropout. The output layer consisted of a single neuron with ReLU activation to ensure non-negative predictions. The model was trained using the Adam optimizer and mean squared error as the loss function.

All models were trained and tested using 10-fold cross-validation. Prediction accuracy was evaluated by calculating the mean and standard deviation of the mean squared error (MSE), mean absolute percentage error (MAPE), the Spearman correlation coefficient between the actual and predicted Hb/kg index values, and the coefficient of determination (R^2).

Table 1. The differences in Hb/kg Index by gender, BMI Groups, type of surgery, CTD groups and association with age

Variables	Groups, mean ± SD			Test and p-value
Gender	Men (n=134, 86.7%)<br>Mean 1.98 ± 0.69	Women (n=61, 23.3%)<br>Mean 1.75 ± 0.76		t-test p=0.044
Age				Pearson correlation coefficient r, p=0.628
BMI Groups	Group I**<br>(n=56, 28.7%)<br>Mean 2.08 ± 0.73	Group II<br>(n=67, 34.4%),<br>Mean 2.06 ± 0.71	Group III**<br>(n=72, 36.9%)<br>Mean 1.64 ± 0.64	ANOVA p <0.001, η^2=0.08; difference between means of Group 1 and Group 3 Scheffe p=0.003
Type of surgery	Surgery Type 1<br>(n=101, 51.8%)<br>Mean 1.99 ± 0.66	Surgery Type 2<br>(n=57, 29.2%)<br>Mean 1.79 ± 0.78	Surgery Type 3<br>(n=37, 18.9%)<br>mean 1.89 ± 0.75	ANOVA p=0.241, η^2=0.015;
CTD_group	CTD Group I<br>(n=145, 74.4%)<br>Mean 1.84 ± 0.72	CTD Group II<br>(n=50, 35.6%)<br>Mean 2.11 ± 0.68		t test p=0.021

Abbreviations: ANOVA – analysis of variance; SD – standard deviation; BMI – body mass index; CTD – chest tube drainage

Table 2. The features selected by Lasso Regression, coefficients and statistical significance.

Variables	Lasso Coefficient	P-Value
BMI	-0.18	<0.001
Hb T_0 (Pre-op, initial)	0.85	<0.001
Hb T_1	-0.69	<0.001
Ht T_{18}	-0.07	0.015

Abbreviations: BMI – body mass index; Hb T_0 – pre-op, initial hemoglobin; HbT_1 – Hb immediately after surgery; HtT_{18} – hematocrit 18 hours after surgery.

Table 3. Selected significant variables based on Spearman rho p<0.05

Variables	Spearman, rho	p-value
BMI, kg/m^2	-0.31	0.000
Hb.T0.CVK	0.37	0.000
Hb.T1.CVK	-0.29	0.000
Hb.T6.CVK	-0.15	0.032
Hb.T18.CVK	-0.28	0.000
RBC.T1.CVK	-0.32	0.000
RBC.T6.CVK	-0.23	0.001

RBC.T18.CVK	-0.34	0.000
Ht.T1.CVK	-0.27	0.000
Ht.T6.CVK	-0.19	0.007
Ht.T18.CVK	-0.34	0.000
aPTT.T18	0.15	0.040
FibrinogenT1	-0.31	0.000
FibrinogenT6	-0.19	0.009
FibrinogenT18	-0.28	0.000
CTD.2h	0.17	0.021
CTD.3h	0.19	0.008
CTD.4h	0.20	0.005
CTD.5h	0.23	0.001
CTD.6h	0.24	0.001
CTD.summary	0.25	0.000
CTD_group	0.18	0.014

Abbreviations: BMI – body mass index; Hb – hemoglobin; RBC – red blood cells; Ht – hematocrit; aPTT – activated partied thromboplastin time; CTD – chest tube drainage; CTD2h – chest tube drainage 2 hours after surgery, CTD3h – 3 hours after surgery, CTD4h – 4 hours after surgery, CTD5h – 5 hours after surgery, CTD6h – 6 hours after surgery, CTD.summary – 18 hours after surgery. CTD Time points: T_0 – before surgery, T_1 – immediately after surgery, T_6 and T_{18} – 6 and 18 hours after surgery

Table 4. Prediction accuracy for different statistical models

Model	MSE Mean ± SD	MAPE Mean ± SD	Corr Mean ± SD	R2 ± SD
LR	0.12 ± 0.13	0.20 ± 0.11	0.90 ± 0.08	0.77 ± 0.17
Lasso	0.15 ± 0.07	0.31 ± 0.17	0.91 ± 0.06	0.68 ± 0.07
Lasso-OLS	0.08 ± 0.04	0.18 ± 0.10	0.92 ± 0.06	0.82 ± 0.13
SVM	0.17 ± 0.08	0.31 ± 0.19	0.83 ± 0.09	0.64 ± 0.15
kNN	0.35 ± 0.14	0.48 ± 0.29	0.58 ± 0.09	0.26 ± 0.12

DT	0.30 ± 0.09	0.33 ± 0.17	0.65 ± 0.19	0.24 ± 0.44
XGBoost	0.15 ± 0.05	0.27 ± 0.12	0.84 ± 0.11	0.65 ± 0.21
DNN	0.34 ± 0.36	0.42 ± 0.35	0.75 ± 0.20	0.42 ± 0.21

Abbreviations: LR – linear regression; Lasso – Lasso regression; Lasso-OLS – Least Absolute Shrinkage and Selection Operator and Ordinary Least Squares; SVM – support vector machine; kNN – k-nearest neighbour classifier; DT – decision tree; XGBoost - extreme gradient boosting; DNN – deep neural network.
